# Perinatal depression and anxiety: screening performance, treatment outcomes and infant-related effects

**DOI:** 10.1192/j.eurpsy.2026.12039

**Published:** 2026-07-31

**Authors:** B. Mihaela, D. Jelaga

**Affiliations:** Department of Mental Health, Medical Psychology and Psychotherapy, Nicolae Testemițanu State University of Medicine and Pharmacy, Chisinau, Republic of Moldova

## Abstract

**Introduction:**

Perinatal depressive and anxiety disorders are common yet underdetected and undertreated, with measurable risks for maternal morbidity and infant outcomes. Despite guideline-endorsed screening, uncertainty remains regarding optimal cutoffs and the real-world effectiveness and safety of psychological/digital and pharmacologic treatments.

**Objectives:**

(1) Quantify screening accuracy for depression/anxiety in pregnancy and ≤12 months postpartum. (2) Estimate treatment outcomes for psychological/digital and pharmacologic options. (3) Synthesize infant-related effects associated with untreated maternal illness and with maternal treatment.

**Methods:**

Databases: MEDLINE/PubMed, Embase, PsycINFO, Web of Science (2020–2025). Designs: cohorts, RCTs, meta-analyses/IPD-MA; prenatal/postpartum. Exclusions: non-perinatal, absent reference/comparator, irrelevant outcomes. Records: 1,204; post-dedup 1,011 screened; full texts 118; included 24; excluded 94. Populations: predominantly women 18-45 y; obstetric/community; sex and mean age not consistently reported. Reference: structured diagnostic interviews where available. Primary metrics: Se/Sp (screening); Hedges g/SMD & response/remission (treatments); RR/OR (infant outcomes).

**Results:**

Screening depression: EPDS ≥11 Se 81%, Sp 88% (AUC ~0.91). PHQ-9 ≥10 Se 84%, Sp 81% (AUC ~0.89); comparable accuracy.

Screening anxiety: postpartum GAD-7 8/9 Se 79.2%, Sp 85.3%; pregnancy EPDS-3A ≥7 Se 61.5%, Sp 86.3% (AUC ~0.82); weaker alone, best adjunct to GAD-7.

Psychological/digital: across RCTs (~6,000), CBT reduced depression (Hedges g ~−0.53 to −0.67; NNT ~4–5). Task-shared delivery: 6-month remission 76.8% vs 47.3% (enhanced usual care; +29.5 pp). ICBT: SMD −0.64 (depression), −0.49 (anxiety) vs controls; attrition +38% (RR 1.38).

Pharmacologic: oral zuranolone (14 days) improved HAMD-17 at Day 15 (MD ~−4.2); response 72% vs 48%, remission 45% vs 23% vs placebo; sustained to Day 45 (remission 53% vs 30%). AEs generally mild–moderate.

Infant-related: untreated prenatal depression associated with higher preterm risk +35–77% (RR 1.35–1.77) and nearly tripled low birthweight (RR 2.98, +198%). Postpartum treatment (SSRIs or psychological care) associated with reduced child externalising symptoms.

**Conclusions:**

Evidence: EPDS (≥11) and PHQ-9 (≥10) provided balanced depression case-finding; for anxiety, GAD-7 at lower thresholds (~8/9 postpartum) outperformed EPDS-3A alone. CBT including task-shared and internet-based formats achieved meaningful improvements; ICBT is scalable but with higher attrition. Short-course zuranolone produced rapid relief with sustained remission to Day 45. Untreated prenatal depression was associated with +35–77% preterm risk and ~3-fold low birthweight, supporting staged screening (depression + anxiety), stepped-care access to CBT/ICBT, and timely pharmacologic therapy. Limitation: heterogeneity in reference standards, cutoffs and follow-up definitions.

**Disclosure of Interest:**

None Declared

